# A Novel Method for Assessing Risk-Adjusted Diagnostic Coding Specificity for Depression Using a U.S. Cohort of over One Million Patients

**DOI:** 10.3390/diagnostics14040426

**Published:** 2024-02-15

**Authors:** Alexandra Glass, Nalander C. Melton, Connor Moore, Keyerra Myrick, Kola Thao, Samiat Mogaji, Anna Howell, Kenneth Patton, John Martin, Michael Korvink, Laura H. Gunn

**Affiliations:** 1 School of Data Science, University of North Carolina at Charlotte, Charlotte, NC 28223, USA; aglass11@charlotte.edu (A.G.); cmoor197@charlotte.edu (C.M.); kthao14@charlotte.edu (K.T.); smogaji@charlotte.edu (S.M.); ahowel31@charlotte.edu (A.H.); kpatto10@charlotte.edu (K.P.); 2 Department of Public Health Sciences, University of North Carolina at Charlotte, Charlotte, NC 28223, USA; nmelton1@charlotte.edu (N.C.M.); kmyrick2@charlotte.edu (K.M.); 3 ITS Data Science, Premier, Inc., Charlotte, NC 28277, USAmichael_korvink@premierinc.com (M.K.); 4 School of Public Health, Faculty of Medicine, Imperial College London, London W6 8RP, UK

**Keywords:** coding specificity, depression, ICD-10, Poisson binomial, principal diagnosis, secondary diagnosis, claims data, risk adjustment

## Abstract

Depression is a prevalent and debilitating mental health condition that poses significant challenges for healthcare providers, researchers, and policymakers. The diagnostic coding specificity of depression is crucial for improving patient care, resource allocation, and health outcomes. We propose a novel approach to assess risk-adjusted coding specificity for individuals diagnosed with depression using a vast cohort of over one million inpatient hospitalizations in the United States. Considering various clinical, demographic, and socioeconomic characteristics, we develop a risk-adjusted model that assesses diagnostic coding specificity. Results demonstrate that risk-adjustment is necessary and useful to explain variability in the coding specificity of principal (AUC = 0.76) and secondary (AUC = 0.69) diagnoses. Our approach combines a multivariate logistic regression at the patient hospitalization level to extract risk-adjusted probabilities of specificity with a Poisson Binomial approach at the facility level. This method can be used to identify healthcare facilities that over- and under-specify diagnostic coding when compared to peer-defined standards of practice.

## 1. Introduction

The International Classification of Diseases (ICD) is a medical coding system that is continuously updated and used to catalog health conditions by categories of similar diseases under more specific conditions [1]. The World Health Organization has been responsible for ICD since 1992, providing a standardized method of recording and tracking diseases worldwide [1]. ICD-10 (10th revision) coding affects healthcare delivery, payments and reimbursements, and disease surveillance. ICD-10-CM, which is the clinical modification (CM) developed and maintained by the Centers for Disease Control and Prevention (CDC) that was introduced shortly after, provided a 400% increase in diagnosis codes while also increasing 18-fold the coding for procedures [2]. This enhancement aimed to add granularity and specificity in clinical records for diagnoses and procedures, though some clinical specialties have been more directly affected than others [1,2,3]. However, a larger catalog of ICD-10 codes does not directly imply widespread use of the more granular coding options [4].

Medical coding is expected to be accurate, complete, and specific to the finest degree possible. Rigorous coding ultimately benefits patients, healthcare providers, and payors [5]. Coding errors can occur when the physician documentation is insufficient [6] or the coding staff is improperly trained. One essential aspect of the coding process is the concept of *coding specificity*, which can be regarded as medical coding to the greatest level of precision supported by a clinical diagnosis code [5]. Unspecified codes should be the last resort when a more specific diagnosis is not viable. The United States (U.S.) Centers for Medicare and Medicaid Services’ (CMS) guidelines indicate that “*When sufficient clinical information is not known or available about a particular health condition to assign a more specific code, it is acceptable to report the appropriate unspecified code*” [7]. While increasing coding specificity rates has been recommended when appropriate [5], the focus on rates alone can be problematic. Rates can be sensitive to hospital volume, patient mix, and other factors, which may result in varying degrees of specificity, whether clinically supported or not. Additionally, coding to the highest degree of specificity is not always clinically justified, such as in instances where a specified secondary diagnosis may not be needed for the provision of treatment for the principal diagnosis during an inpatient or emergency stay or when resources are not available to provide that additional level of specificity [8,9,10].

In the inpatient setting, coding specificity is the responsibility of the provider and coder, who must work together to record a detailed clinical description of the diagnosis or procedure [5,11]. Accurate levels of diagnostic coding specificity, when possible, help align reimbursements with healthcare costs and provide patients with accurate medical records to more effectively guide treatment plans. Detailed documentation and coding of diagnoses influence reimbursement but also represent an additional cost for healthcare providers and payors. Higher degrees of coding specificity, especially upon introducing ICD-10-CM, require additional levels of expertise among coders [5], which may be a larger burden in facilities providing more general services or those with limited resources or personnel. Practices may also suffer financial loss if coding specificity is insufficient or inappropriate [5]. In some cases, payors may determine that codes lacking specificity are used improperly (or overused), potentially leading to a denied claim [5,7]. Conversely, over-specificity, when not clinically warranted, is problematic, as such coding may overstate patient care needs, unduly inflate reimbursement through Diagnosis Related Group (DRG) creep, and exaggerate a patient’s clinical risk.

Despite the importance of accurate coding for patient conditions, enforcing and maintaining a high, yet appropriate, level of coding specificity has remained an issue since the inception of the ICD-10 system [5,11]. While studies have been conducted to identify sources of coding errors throughout a patient encounter or episode, there is minimal literature examining how best to quantify coding specificity as an independent metric or how to predict or identify where unspecified codes are (or have the potential to be) most misaligned with the patient’s true diagnosis, that is, when the diagnosis may be accurate but the level of coded specificity is not appropriate for the clinical diagnosis [11,12]. Even in studies where coding specificity is observed or utilized as an analytical component, the methods used to develop the specificity metric are often vague, overlooked entirely, and/or narrowly defined in a disease-specific form, resulting in additional complexity to generalize across conditions [13,14,15]. The lack of standardized methods for measuring, quantifying, and analyzing coding specificity represents a significant gap in knowledge for the healthcare community.

While similar methodologies have been developed in the literature for measuring coding intensity [16,17], we aim to create a metric by which to measure and risk-adjust coding specificity that would allow for comparative analysis of facilities, thus identifying where coding specificity may need improvement against healthcare industry standards or aspirational peers. A metric that could have potential for widespread implementation would not require clinical inputs regarding appropriateness of specificity, which may not be agreed upon across physicians, change substantially over time, and be costly to obtain and maintain, as well as being less generalizable across health conditions. Such metric should also be relatively easy to implement without major costs and with readily available patient and facility data, such as administrative claims data, though sufficiently flexible to account for other information when available.

Depression, affecting 18.5% of the U.S. adult population in 2020 [18], has been identified as one of the conditions that is commonly reported with unspecified diagnosis codes [5]. Three of the most common codes produced by the ICD-10 criteria for depression include: major depressive disorder (F32); dysthymic disorder (F34.1); and unspecified depression (F32.A) [5,19,20]. ICD-10 codes related to depression are also grouped within the DRG list of depressive neuroses (DRG 881) [20]. Recommendations for an initial diagnosis using ICD-10 codes require identifying five symptoms of depression lasting two weeks or more and must include depressed mood or loss of interest [19,21]. However, depression should only be considered after accounting for the absence of medical conditions that can mimic symptoms of depression (e.g., thyroid problems or brain tumors) and after ruling out bereavement or sadness caused by life-altering events [21].

The degree to which coding specificity varies across providers for depression patients remains unclear. Facilities do not have a standard against which they can measure their levels of coding specificity of depression diagnoses during inpatient hospitalizations, particularly because of potential case mix differences across facilities. This calls for a method that risk-adjusts for such differences and provides an objective and standardized metric against which each facility can measure variation in coding specificity. This study aims to demonstrate a novel approach for measuring the risk-adjusted probability of coding specificity controlling for patient and facility characteristics, both across principal and secondary diagnoses of depression, while building an aggregated metric that can be used at coarser levels. Such an approach can be used by quality control personnel to enhance standards of practice around coding specificity, not only for individuals diagnosed with depression but also across a wide spectrum of health conditions.

## 2. Materials and Methods

Data were obtained from the Premier Healthcare Database (PHD), a national, hospital-based, service-level, and private all-payor database that contains information on inpatient discharges [22]. The analysis comprises *N* = 1,071,575 observations of acute inpatient hospitalizations of first-patient stays with discharge dates in 2022 with an identified principal or secondary diagnosis of depression. Specificity for a depression principal diagnosis was identified, and, when multiple depression secondary diagnoses occurred, specificity for the secondary diagnosis was defined as specificity for at least one of these depression secondary diagnoses. The ICD-10 codes defining the patient cohort consisted of the F32 (depressive episode) and F33 (major depressive disorder, recurrent) codes.

The data consist of the following information, in addition to masked patient and facility identifiers: (1) binary response variables representing specificity of principal and secondary diagnoses of depression; (2) patient characteristics, which include age, sex, race, length of stay (log-transformed due to its large right-skewness), primary payor, point of origin, discharge status, count of procedures performed during the inpatient stay, CMS fiscal year indicator, five county-level Agency for Toxic Substance & Disease Registry (ATSDR) social vulnerability indices (SVIs) [23], COVID-19 indicator, and Medicare Severity (MS)-DRG type; and (3) facility characteristics including teaching status, academic status, urban/rural status, ownership status, bed count, hospital-level case mix index (CMI), and state. The primary payor variable refers to the insurance provider that assumes the primary responsibility for covering the costs of a healthcare claim. For example, “Medicare traditional” indicates that the patient is covered under Medicare, the U.S. government’s insurance plan for patients aged 65 or older, while “Medicaid traditional” refers to the U.S. government’s insurance plan for low-income patients and their families.

Descriptive statistics were calculated for all aforementioned variables, including means/counts and standard deviations/percentages. Categories with low counts and similar meanings (e.g., charity and indigent primary payor types) or adjacent ordered categories (e.g., ages 0–9) with low counts were grouped together.

Univariate and multivariate logistic regression analyses were used to extract associations between patient-level and facility-level variables and the coding specificity of depression principal and secondary diagnoses. Odds ratios (ORs), 95% confidence intervals (CIs), and *p*-values were calculated and tabulated for all four analyses. The receiver operating characteristic (ROC) curve was constructed, and the corresponding area under the curve (AUC) was computed for both the depression principal and secondary diagnosis specificity multivariate logistic regression models.

The unknown probability ${(\pi }_{p,f})$ of coding specificity of principal, or secondary, diagnosis for patient hospitalization *p* in facility *f*${(S}_{p,f})$ was modeled with a multiple logistic regression including covariates ($X_{p,f}$) that represent both patient and facility characteristics using the equation below:

(1) $$\mathrm{logit}\;(\pi_{p,f}[S_{p,f}|X_{p,f}])=\alpha +\beta^{T}X_{p,f},$$

where *α* is the intercept and the vector $\beta^{T}$ contains corresponding regression coefficients for $X_{p,f}$. Each patient hospitalization’s coding specificity within a facility *f* was assumed to be independently distributed with an unequal, unknown probability $\pi_{p,f}$. Since the coding specificity events were not identically distributed, this total count for each facility *f* ($i.e.,\sum_{p\subset f}S_{p,f})$ was assumed to follow a Poisson Binomial (PB) distribution with a probability vector ${\hat{\pi }}_{p\subset f}$, composed of the probabilities for each patient hospitalization *p* within facility *f* (i.e.,p⊂f). Upon extracting the estimated probabilities ${\hat{\pi }}_{p,f}$ for each patient hospitalization *p* and facility *f*, these were used to assess whether each facility’s total count of specified diagnoses was under-, in line with, or over-specified compared with their healthcare industry peers via a user-defined probability threshold *t*.

Without loss of generality, we applied a common threshold *t* = 0.025 to identify facilities operating outside peer standards’ confidence bounds (2.5th and 97.5th percentiles) denoted by $Q_{L}$ and $Q_{U}$, representing under and over specificity, respectively, for facility *f*:

(2) $$P(\sum_{p\subset f}S_{p,f}\sim PB[\{{\hat{\pi }}_{p\subset f}\}]<Q_{L})=t$$

(3) $$P(\sum_{p\subset f}S_{p,f}\sim PB[\{{\hat{\pi }}_{p\subset f}\}]>Q_{U})=t$$

Visualizations of the facility-specific metrics were produced to demonstrate under-specifying (*p* < 0.025) and over-specifying (*p* > 0.975) facilities using the cumulative distribution function of the facility-specific Poisson Binomial distribution and the observed specificity count across patient hospitalizations for that facility. Geospatial U.S. maps of adjusted odds ratios of coding specificity by state were also produced across both outcomes, with New York selected as the reference state based on its largest healthcare expenditure (per capita) in the U.S. [24].

## 3. Results

Table 1 provides descriptive statistics for all variables across *N* = 1,071,575 unique inpatient hospital admissions where depression was recorded as the principal or secondary diagnosis. Of these hospitalizations, 16,437 had depression as a principal diagnosis. Of the principal diagnoses, 4736 (28.8%) were coded as unspecified.

Most of the patients were aged 65 to 69 years old (12%), female (65%), and identified as White (80%). The median length of stay was 4 days, the average number of procedures per hospitalization was 2.9 (SD 2.7), and most hospitalizations occurred in the CMS 2022 fiscal year (75%). Traditional Medicare was the most common primary payor (29%), the most common point of origin was a non-healthcare facility (80%), and most patients were discharged to home or self-care (53%). Average scores for patients’ SVI values were 0.53 (SD 0.26) for socioeconomic status, 0.51 (SD 0.25) for household characteristics, 0.66 (SD 0.24) for racial and ethnic minority status, 0.60 (SD 0.25) for housing type and transportation, and 0.58 (SD 0.25) for overall vulnerability. Seven percent of patients experienced COVID-19 during their hospitalization, and 74% of patients had a medical MS-DRG type.

Most of the hospitals were non-teaching (73%), non-academic (83%), located in an urban setting (88%), voluntary non-profit private (65%), and had more than 400 beds (43%). The average patient case mix index was 1.7 (0.29). Data were collected from facilities in all fifty states, but the five states with the largest numbers of observed hospitalizations were Florida (9%), New York (7%), Texas (6%), North Carolina (6%), and Ohio (5%).

Table 2 contains the univariate and multivariate logistic regression results, including odds ratio estimates, 95% confidence intervals, and *p*-values for the specificity of a principal depression diagnosis. Patient characteristics such as age, primary payor, and SVI had a significant association with depression principal diagnosis coding specificity across multiple categories based on the multivariate logistic regression model. The odds of depression principal diagnosis coding specificity were at least 46% higher among patients aged less than 80 years, with the exception of those less than 10 years old, when compared to patients 85+ years old (OR ≥ 1.459; *p* ≤ 0.041). Males experienced 24% lower odds of depression principal diagnosis specificity compared to females (OR = 0.76; *p* < 0.001). No significant differences in odds of specificity were found by race upon accounting for all other factors. However, every additional unit in the Racial and Ethnic Minority Status SVI was associated with approximately 49% lower odds of specificity (OR = 0.506; *p* = 0.003). Length of stay (log-transformed) was also positively associated with higher odds of principal diagnosis specificity (OR = 1.82; *p* < 0.001). Differences were found across some categories of primary payor, point of origin, and discharge status. However, there was no significant association between COVID-19 status, CMS fiscal year period, count of procedures, or other SVI measures with depression principal diagnosis specificity. Patients grouped with a surgical MS-DRG type experienced substantially lower odds of depression-related principal diagnosis specificity (OR = 0.288; *p* < 0.001) when compared to those with a medical MS-DRG.

Patients attending rural facilities did not experience statistically different odds of specificity of a depression principal diagnosis compared to those attending urban facilities (OR = 1.009; *p* = 0.917). Patients attending teaching facilities experienced lower odds of depression principal diagnosis specificity (OR = 0.680; *p* < 0.001), whereas those attending facilities with an academic status experienced higher odds of depression principal diagnosis specificity (OR = 1.465; *p* = 0.001). All significant ownership categories were associated with lower odds of depression principal diagnosis specificity compared to the reference category (voluntary nonprofit private). No clear pattern emerged by bed size, and the case mix index was found to be non-significantly associated with principal diagnosis coding specificity. However, substantial differences were detected by state when compared to New York as the reference state. For example, states like California experienced much higher odds of specificity of a depression principal diagnosis (OR = 1.995; *p* < 0.001), while others like New Jersey experienced substantially lower odds of principal diagnosis specificity (OR = 0.247; *p* < 0.001).

Table 3 contains the univariate and multivariate logistic regression results, including odds ratio estimates, 95% CIs, and *p*-values, for the specificity of depression-related secondary diagnoses. The multivariate analysis demonstrates that individuals of all age groups experienced significantly higher odds of specificity of depression secondary diagnoses compared to those 85 and older (OR ≥ 1.116; *p* ≤ 0.036). Black individuals experienced 12.5% lower odds of secondary diagnosis specificity than White patients (OR = 0.875; *p* < 0.001). Males experienced approximately 5% higher odds of secondary diagnosis specificity than females (OR = 1.054; *p* < 0.001). Length of stay (log-transformed) was also positively associated with higher odds of depression secondary diagnosis specificity (OR = 1.237; *p* < 0.001). Primary payor type, point of origin, and discharge status all contained categories with statistically significant associations with the outcome. Patients who experienced larger numbers of procedures also experienced higher odds of secondary diagnosis specificity (OR = 1.007; *p* < 0.001). Those discharged in the 2023 CMS fiscal year experienced 4.2% increased odds of depression secondary diagnosis specificity (OR = 1.042; *p* < 0.001). All SVI indices were also significant, as was COVID-19 status, with COVID-19-positive patients experiencing approximately 7% lower odds of depression secondary diagnosis specificity (OR = 0.929; *p* < 0.001). Patients with a surgical MS-DRG also experienced 14.5% lower odds of depression secondary diagnosis specificity compared to those with a medical MS-DRG type (OR = 0.855; *p* < 0.001).

Patients admitted to teaching facilities experienced significantly higher odds of depression secondary diagnosis specificity (OR = 1.177; *p* < 0.001), while those attending academic facilities experienced lower odds of secondary diagnosis specificity (OR = 0.790; *p* < 0.001). Rural facilities provided higher odds of specificity to their patients (OR = 1.409; *p* < 0.001). Some differences were found by ownership status, and patients attending facilities with lower bed counts had lower odds of depression secondary diagnosis specificity compared to those with over 400 beds (OR ≤ 0.937; *p* < 0.001). Patients attending hospitals with larger case mix index values were associated with lower odds of secondary diagnosis specificity (OR = 0.873; *p* < 0.001). Finally, substantial state-based differences were detected, with most states experiencing higher odds of depression secondary diagnosis specificity than NY. For example, individuals in states like MN experienced substantially larger odds of depression secondary diagnosis specificity compared to NY (OR = 11.255; *p* < 0.001).

**Figure 1:**
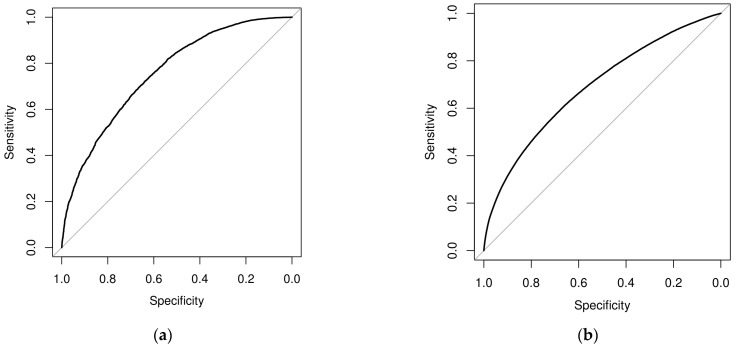
Receiver operating characteristic (ROC) curves of specificity of a depression-related principal diagnosis (**a**) and secondary diagnosis (**b**) using the multivariate logistic regression model.

Figure 1 contains the ROC curves resulting from the multivariate logistic regression analyses of the coding specificity of the principal (a) and secondary (b) diagnoses of depression. The corresponding AUC values were 0.7555 and 0.6874, respectively, indicating a slightly better fit for the model assessing the specificity of a depression principal diagnosis.

**Figure 2:**
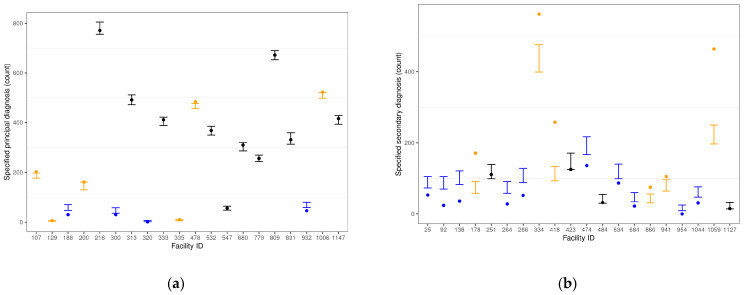
Observed counts of specificity of depression principal (**a**) and secondary (**b**) diagnoses by facility (dots) for two samples of 20 facilities, together with 95% confidence intervals based on the Poisson Binomial model. Facilities that under-specify depression diagnoses compared to healthcare industry peers are depicted in blue (*p* < 0.025), while those that over-specifying depression diagnoses compared to peers are depicted in orange (*p* > 0.075). Facilities that specify depression diagnoses in line with peers are depicted in black.

Figure 2 contains a visual representation of the use of the Poisson Binomial metric for identification of facilities’ specificity of depression principal (a) and secondary (b) diagnoses against healthcare industry peers upon adjusting for patient and facility characteristics. A sample of 20 facilities is portrayed in each plot, with colors denoting coding specificity performance versus peers. Observed counts below the 95% CIs identify facilities that under-specify depression diagnoses compared with their peers (blue), while observed counts above the 95% CIs identify facilities that over-specify depression diagnoses versus peers (orange). Finally, those depicted in black represent facilities that specify depression diagnoses in line with their healthcare industry peers.

**Figure 3:**
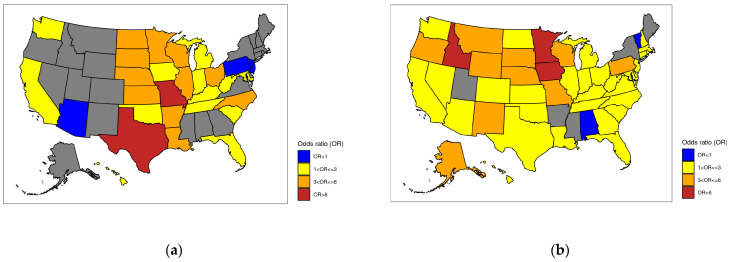
U.S. map representing the adjusted odds ratios, by state, of specificity of depression-related principal (**a**) and secondary (**b**) diagnoses against the reference state of New York. Non-significant adjusted odds ratios are represented in gray. Under-specificity is represented in blue, while over-specificity is clustered across three different groups (yellow, orange, and brown) based on adjusted odds ratio ranges.

Finally, Figure 3 contains U.S. maps representing adjusted odds ratios for the two outcomes. States portrayed in grayscale represent those in which patients have similar odds of coding specificity of depression diagnoses compared with the reference state (New York). States where the odds of diagnosis specificity are below those of New York are represented in blue, while the other color scales represent different degrees of state-level over-specificity of depression diagnoses (see Figure 3 legend). Both maps indicate that New York is generally underspecified across both principal and secondary depression diagnoses when compared to most states.

## 4. Discussion

We propose a two-step approach for modeling coding specificity at the facility level. First, a multivariate logistic regression model is proposed to measure, at the patient hospitalization level, the association between the coding specificity of principal and secondary diagnoses of depression and a set of patient- and facility-level characteristics. In a second step, a Poisson Binomial approach builds upon the risk-adjusted logistic-derived patient-level specificity probabilities to estimate the anticipated 95% confidence interval for coding specificity counts per facility across patient hospitalizations if facilities were to operate in line with healthcare industry standards. Over- and under-specifying facilities are then identified upon comparing their observed coding specificity counts across patient hospitalizations and the aforementioned 95% confidence intervals. We then visualize the facility-specific metrics to demonstrate under- and over-specifying facilities. While outside the scope of this manuscript, facilities can also be ranked by risk-adjusted specificity since the *p*-value-based metric already adjusts for both size (i.e., counts) and strength of evidence.

Patient characteristics were associated with the coding specificity of both the principal and secondary diagnoses. Higher odds of specificity for both principal and secondary diagnoses were generally associated with lower ages compared with those 85+ years old. This may be related to a larger complexity in diagnosis or the presence of more comorbidities. However, it could also be related to a lower quality of coding and/or care provided to older populations [25]. Race was not associated with differences in odds of specificity, with the exception of the secondary diagnosis, where Black patients experienced substantially lower odds of coding specificity, which may relate to differences in coding practices by practitioners and/or differences in information-seeking behaviors by patients [26]. This could reflect findings in prior research showing that disparities in the treatment of depression by race/ethnicity among older adults may still be present [27]. Males experienced substantially lower odds of principal diagnosis specificity but higher odds of secondary diagnosis specificity for depression compared to females. It is unclear whether this is confounded by other factors, such as age, due to differentials in life expectancy and sex-related imbalances in the age-sex pyramid, particularly in the U.S. [28].

Patients with longer stays experienced higher levels of specificity in both principal and secondary diagnosis. This could be due to the additional time and resources employed during the inpatient stay or as a result of the complexity of their cases. Clinicians may spend less time documenting patients with shorter stays. Some differences were observed by the primary payor. However, the patient mix by payor could also be heterogeneous. For example, those with employer contracts as the primary payor may be experiencing higher odds of principal and secondary diagnosis coding specificity because they are a younger population than those receiving healthcare through Medicare, which is the reference category, though it could also relate to requirements related to worker’s compensation. Some social vulnerability indices were also related to differing degrees of coding specificity. However, the information content in this variable likely overlaps with other variables such as age and race/ethnicity. Patients grouped with a surgical MS-DRG experienced lower odds of principal and secondary diagnosis specificity when compared to those with a medical MS-DRG. One possible explanation for this discrepancy is that surgical patients may receive a principal diagnosis that is primarily focused on their surgical condition, which can overshadow or lead to a less detailed assessment and diagnosis of mental health conditions such as depression. Surgical patients who undergo a range of medical tests and evaluations specific to their surgical procedures may experience a more limited extent to which mental health concerns are addressed and documented as the principal diagnosis during the inpatient hospitalization. Additionally, those performing surgical procedures who may be responsible for the patient during inpatient stay may not be the same physicians identifying and/or treating any underlying depression diagnosis. Multiple procedures may require increased attention and precision, leading to more detailed physician consultations and billing practices that may impact coding specificity.

Facility-level characteristics were also associated with the specificity of both principal and secondary diagnosis. However, differences by diagnosis type were found. For example, patients who attended teaching facilities experienced lower odds of principal diagnosis specificity yet higher odds of secondary diagnosis specificity. However, the reverse is seen in academic status. This could relate to high levels of collinearity affecting some of the facility-level variables, so cautious interpretation is advisable. Facilities’ case mix index was significantly associated with lower odds of specificity for both types of diagnoses. This indicates that hospitals dealing with more complex cases tend to underspecify in terms of depression diagnoses. This could relate to the severity of cases and the potential need to allocate resources unevenly across health conditions. Substantial differences were observed by state, with the odds of coding specificity higher across multiple states when compared to NY. This again could reflect differences in patient composition or complexity by state, but also the variability in spending per capita, price levels, overall healthcare affordability, and differences in uptake of Medicaid by state [29].

While of some interest, the ultimate purpose of this study is not to explore associations between these patient and facility characteristics and coding specificity outcomes but to leverage them to build a risk-adjusted estimate of the probability of coding specificity that can be used to evaluate facilities’ standards of practice. The purpose of the multivariate logistic regression is to capture the probability of specificity, and the combination of patient and facility characteristics led to high levels of explanatory power, even when a large number of clinical factors were not included in this study. The AUC was 0.76 and 0.69 for the principal and secondary diagnosis specificity models, respectively. It would be reasonable to expect that principal diagnoses are specified at a higher level, since secondary diagnoses could be very unrelated to the primary reason for the inpatient hospitalization, and an accurate diagnosis may not be needed to treat the patient’s condition. However, good levels of explanatory power were also found among patient and facility characteristics for the secondary diagnoses model, which comprises a larger number of individuals between the two analyses. This explanatory power was achieved with relatively low levels of clinical information about the patient. Additional variables describing the clinical characteristics of the patient hospitalization are likely to enhance the AUC levels substantially more.

The AUC values across both types of diagnoses highlight that risk-adjustment of specificity outcomes is important when evaluating hospital coding specificity performance. Otherwise, facilities could be unfairly compared and evaluated. For example, a hospital treating a large population of younger patients may demonstrate high levels of overall coding specificity while actually providing low levels of risk-adjusted specificity. Risk-adjustment allows practitioners to adjust for industry-level differences, while it also allows policymakers to explore whether such differences are warranted or demonstrate disparities or inappropriate standards of practice at the industry level that need to be addressed.

Upon risk-adjusting for patient and facility characteristics, we demonstrate that substantial differences in coding specificity by facility still remain. These differences are more likely to be due to idiosyncrasies and facility-specific processes and practices. We demonstrate these differences in risk-adjusted specificity with a sample of facilities. Our proposed metric can help identify facilities that, upon adjusting for common factors that affect variability in coding specificity, still perform substantially away from common healthcare practice.

From a practical standpoint, the model outcomes can serve multiple purposes toward enhancing clinical data abstraction, such as: (1) Serve as flags for facilities that, upon risk-adjusting for their patient mix, may be operating at standards that widely differ from those of their peers. This can take the form of under-specificity or over-specificity; (2) Serve as an intra-facility flag for physicians or units who may also be under- or over-specifying when measured against peers, which may be internal or external to the facility; (3) Serve as an intra-facility flag for specificity practices across health conditions; and (4) Serve to measure the clinical abstractors themselves to conduct practical root cause analysis. In all cases, the actionable steps from flagging such differences in operations against peers could be a more in-depth gathering of information as to whether diagnoses are insufficiently precise, personnel may not be sufficiently versed in the granularity offered in ICD-10 codes, or clinical abstraction may be enhanced (e.g., due to insufficient or incorrectly recorded clinical diagnoses), or whether the diagnoses are overly precise given the information within the respective clinical records. Our approach can be applied across health conditions and units, thus serving as an automated and low-cost first-warning system for coding specificity practices. Thus, both quality-control personnel within the facilities and outside of them (e.g., claims personnel) can assess coding practices that may depart from standard practice, with or without cause, and without the need for a full clinical assessment across patients, which would be substantially more costly. While false positives may occur and coding specificity practices may be warranted on a clinical basis, this approach can serve to identify facilities, units, or physicians most likely to be true positives (intended or unintended) and who may be departing from such practices in ways that may need to be addressed. Ultimately, this would result in a benefit for both the facilities and patients, enhancing the quality of medical records and identifying and resolving inefficiencies where present. Facilities could benefit from the maximization of reimbursement (when under-specifying) and the minimization of risks (e.g., reputational or financial) due to over-specification [2,5].

As the U.S. and other countries look toward the implementation of ICD-11, standardized methods to measure variation in coding, such as those proposed here, will have an important role in providing hospitals with a fair benchmark against which coding practices can be evaluated. Since it is unclear whether there may already exist coding specificity differences between the U.S. and other countries due to the lack of literature, further studies are needed across healthcare delivery systems to assess whether the findings in our study also apply to systems that may be more centralized, such as the United Kingdom’s National Health Service. The effect of the changes from ICD-10 to ICD-11 on such potential differences across healthcare systems is also unclear. However, our model allows for a rolling estimation of specificity levels. Thus, the impact of interventions, such as those derived from quality-control actions or from transitions from ICD-10 to ICD-11, could be measured with approaches such as interrupted time series analyses.

While our approach does not provide a raw measure to define ‘correct’ levels of coding specificity, it provides the user with a peer-based metric. Institutions that aspire to perform in line with industry standards (or standards defined by a subset of peers) can compare themselves with these standards through the counterfactual outcomes of this model. To our knowledge, the approach demonstrated in this manuscript is the first to address, in a fully extrapolatable way, the issue of diagnostic coding specificity in a large, population-based study. Finally, while our approach is built on a logistic regression model, alternative approaches are possible. We proposed a logistic regression approach due to the additional interpretability of the intermediate model outcomes. Also, this approach is useful as it serves as a natural intermediate outcome (estimated probability of specificity) for grouping/clustering across hospitalizations that share common underlying traits, such as hospitals, physicians, states, or any other clustering variable. However, other artificial intelligence/supervised learning approaches may be better suited when predictability at the hospitalization level is more relevant than the analysis of coding specificity practices.

### Strengths and Limitations

Claims data are generally more readily available and standardized to a greater degree than medical records, allowing for a larger observation cohort and greater generalizability of methods and results across diseases/patient cohorts. Our cohort, which is comprised of over one million observations, is, to our knowledge, the largest cohort in the literature for measuring and modeling the coding specificity practices of any disease. The primary limitation of relying on claims data are a lack of patient-level clinical data that would be contained in an electronic health record (EHR) or similar medical record. Clinical factors such as patient underlying health conditions, severity of patient health concerns, or whether procedures are urgent or elective likely play a role in the way patient diagnoses are coded and would serve to improve the robustness of our evaluation metrics. However, the information contained in our claims data are sufficient to develop a metric by which to evaluate coding specificity, including patient- and facility-level characteristics, and would only be improved by this additional information when it is available. Also, limiting a model to only be usable when such EHRs are available would hamper its practical utility. Some variable categories were also grouped due to low value counts (e.g., ages 1–4 and 5–9 combined into a single 0–9 category), but arguably some of these groupings could be deemed subjective. Regardless, their impact on the results is unlikely to be relevant, especially given the very low counts as a proportion of the overall sample size.

The facility type and distribution of physician specialties are not considered but could be relevant factors. Facilities that provide healthcare across a wide range of health conditions may not have the level of specialization among their physicians and coders compared with those in more specialized facilities.

Race was included to measure potential inequity of care (i.e., coding) and to demonstrate the approach in general terms among practitioners. However, the inclusion of this variable in the construction of risk-adjusted metrics continues to be a debatable topic, with practitioners still using it to guide clinical decision-making [30]. Because of the nature of this ongoing debate between recommendable and currently implemented practice, the variable was included to demonstrate differences by race, whether warranted by clinical diagnosis or not. The model can easily be adapted to exclude race and cluster practices, thus demonstrating differences by race and practice (or grouping across practices by race). This is outside the scope of this study and would be future research.

While a facility may contain multiple hospitalizations per patient, we restricted our dataset to one (specifically their first with a 2022 discharge date) hospitalization per patient to avoid excessive influence by patients who may have large numbers of inpatient stays due to recurring needs. This exclusion helped mitigate concerns that subsequent stays would no longer be independent hospitalizations. This cohort definition can be relaxed by including additional patient hospitalizations and random effects per patient, or by including a factor to account for second or later hospitalizations. However, the computational complexity and burden of such an approach should also be considered, as well as the heterogeneity of such a population. Ultimately, coding specificity during the first inpatient stay may likely be a lower bound for the specificity of further inpatient stays with the same diagnoses if adequate records are maintained and clinical staff carefully review them, thus providing a conservative metric for each facility. At the facility level, random effects could be used for facilities; however, this would increase the computational complexity substantially.

Multicollinearity was observed for several variables, both at the patient and facility levels, so caution is recommended when drawing conclusions about individual variable relevance (or directionality of any association) for risk adjustment. However, to account for this limitation, we also performed univariate analyses in addition to multivariate analyses, providing additional information to measure variable associations. It is also important to note that this multicollinearity does not impact overall model performance or the development of a metric to assess variations in coding specificity. The multivariate models’ AUCs and the subsequent Poisson Binomial metrics would not be affected by multicollinearity, and, therefore, the model is flexible enough to be expanded with additional variables, if available, even if highly correlated with existing ones. Also, state-level clustering of facilities was not considered in this study, where hospitals may be part of a shared health system using centralized teams of coders or commonly defined standards. This may result in inter-facility correlations. In this case, the borrowing of information across facilities could be explored, though outside the scope of this study.

Observations are likely not independent since common latent factors could exist. For example, shared coders or physicians who may operate across facilities could breach the assumption of independence. Also, facilities may have common ownerships, which, in turn, could lead to similar standards of practice. However, these issues do not invalidate the methodology proposed. The grouping was demonstrated at the facility level, but it could be performed at any level, including at the physician or facility owner levels.

The definition of the secondary diagnosis was made to reflect any specified secondary diagnosis of depression. However, when multiple secondary diagnoses of depression are present, this binary definition could be subjective. Regardless, only a small number of hospitalizations reflected multiple secondary diagnoses of depression, and an analysis using an alternative definition of ‘all specified diagnoses of depression’ as the outcome rendered very small differences in AUC.

## 5. Conclusions

This study aims to demonstrate a novel approach for measuring the risk-adjustment specificity controlling for patient and facility-level characteristics for principal and secondary diagnoses of depression. This approach is extended to create an aggregate metric that can be used at coarser levels, grouping by any observable common factor, and demonstrated at the facility level. In this study, we propose a multivariate logistic regression model for coding the risk-adjusted specificity of depression principal and secondary diagnoses. Our findings demonstrate that both patient and facility characteristics commonly available in claims data are relevant to explaining variability in the coding specificity of both the principal and secondary diagnoses of depression. This approach represents one of the building blocks for designing a risk-adjusted, facility-specific index that can be used by quality control personnel to compare facilities’ coding specificity practices with peers across diseases. While we demonstrate our novel approach with a large patient cohort diagnosed with depression during hospitalizations, the method can be applied to any disease cohort and any grouping-level variable. Therefore, our approach fills a gap in the already scarce literature on coding specificity.

## Figures and Tables

**Table 1:** Summary statistics, including counts (%) and means/proportions (standard deviations [SD]) of study outcomes as well as patient- and facility-level characteristics.

Study Variables	Count or Mean/Proportion (% or Standard Deviation [SD])
**Outcomes**	
Specificity of the depression principal diagnosis (count, proportion)	11,701 (71%)
Specificity of depression secondary diagnoses (count, proportion)	80,116 (8%)
**Patient Characteristics**	
Age (Years)	
0–9	208 (<1%)
10–14	5408 (1%)
15–19	16,017 (1%)
20–24	28,158 (3%)
25–34	86,719 (8%)
35–44	90,578 (8%)
45–54	120,430 (11%)
55–59	91,701 (9%)
60–64	114,939 (11%)
65–69	124,098 (12%)
70–74	123,580 (12%)
75–79	108,929 (10%)
80–84	77,339 (7%)
85+	83,471 (8%)
Sex	
Female	698,038 (65%)
Male	373,537 (35%)
Race	
Asian	11,726 (1%)
Black	118,837 (11%)
Other	63,219 (6%)
Unable to determine	24,007 (2%)
White	853,786 (80%)
Log (Length of Stay) (Days) (mean, SD)	1.4 (0.87)
Primary Payor	
Charity/Indigent	2068 (<1%)
Commercial indemnity	59,773 (6%)
Direct employer contract	3106 (<1%)
Managed care capitated	2466 (<1%)
Managed care non-capitated	143,667 (13%)
Medicaid-managed care capitated	22,545 (2%)
Medicaid-managed care non-capitated.	120,312 (11%)
Medicaid traditional	57,926 (5%)
Medicare-managed care capitated.	38,172 (4%)
Medicare-managed care non-capitated.	251,837 (24%)
Medicare traditional	312,783 (29%)
Other	11,980 (1%)
Other government payors	21,483 (2%)
Self-pay	21,213 (2%)
Workers compensation	2244 (<1%)
Point of Origin	
Clinic	83,151 (8%)
Court/Law enforcement	1330 (<1%)
Information not available	14,016 (1%)
Non-healthcare facility	856,254 (80%)
Other	859 (<1%)
Transfer from ambulatory surgery center	1187 (<1%)
Transfer from department unit in same hospital, separate claim	5690 (1%)
Transfer from health facility	12,915 (1%)
Transfer from hospice and under hospice	94 (<1%)
Transfer from hospital (different facility)	68,715 (6%)
Transfer from SNF ^1^ or ICF ^2^	27,364 (3%)
Discharge Status	
Acute inpatient readmission	2038 (<1%)
Discharged to home health organization	171,209 (16%)
Discharged to home or self-care	571,921 (53%)
Discharged to hospice-home	13,897 (1%)
Discharged to hospice-medical facility	14,015 (1%)
Discharged/Transferred to another rehab facility	33,314 (3%)
Discharged/Transferred to cancer center/children’s hospital	292 (<1%)
Discharged/Transferred to court/law enforcement	2061 (<1%)
Discharged/Transferred to critical access hospital	196 (<1%)
Discharged/Transferred to federal hospital	307 (<1%)
Discharged/Transferred to ICF ^2^	8244 (1%)
Discharged/Transferred to long-term care hospital	6954 (1%)
Discharged/Transferred to nursing facility	1394 (<1%)
Discharged/Transferred to other facility	16,660 (2%)
Discharged/Transferred to other health institute not in the list	2823 (<1%)
Discharged/Transferred to psychiatric hospital	16,276 (2%)
Discharged/Transferred to SNF ^1^	153,708 (14%)
Discharged/Transferred to swing bed	2706 (<1%)
Expired	28,121 (3%)
Information not available	3794 (<1%)
Left against medical advice	21,555 (2%)
Still a patient, expected to return	90 (<1%)
Count of Procedures (mean, SD)	2.9 (2.7)
CMS ^3^ Fiscal Year	
2022	804,999 (75%)
2023	266,576 (25%)
Social Vulnerability Indices (mean, SD)	
Household characteristics	0.51 (0.25)
Housing type and transportation	0.60 (0.25)
Overall	0.58 (0.25)
Racial and ethnic minority status	0.66 (0.24)
Socioeconomic status	0.53 (0.26)
COVID-19 Status	
Not identified	994,349 (93%)
Positive	77,226 (7%)
MS-DRG ^4^ Type	
Medical	794,118 (74%)
Surgical	277,407 (26%)
Unknown	50 (<1%)
**Facility Characteristics**	
Teaching Status	
No	780,718 (73%)
Not available	15,313 (1%)
Yes	275,544 (26%)
Academic Status	
No	885,959 (83%)
Yes	185,616 (17%)
Rural/Urban Status	
Rural	132,668 (12%)
Urban	938,907 (88%)
Ownership	
Government—federal	1439 (<1%)
Government—hospital district/authority	62,451 (6%)
Government—local	34,529 (3%)
Government—state	10,281 (1%)
Not available	4771 (<1%)
Physician	1135 (<1%)
Proprietary	43,045 (4%)
Voluntary non-profit—church	156,542 (15%)
Voluntary non-profit—other	61,799 (6%)
Voluntary non-profit—private	695,583 (65%)
Bed Count	
1–50	27,588 (3%)
51–100	52,634 (5%)
101–150	76,610 (7%)
151–200	71,842 (7%)
201–250	96,827 (9%)
251–300	105,830 (10%)
301–350	106,327 (10%)
351–400	75,304 (7%)
>400	458,613 (43%)
Hospital Case Mix Index (mean, SD)	1.7 (0.29)
State Abbreviation	
AK	460 (<1%)
AL	7755 (1%)
AR	11,427 (1%)
AZ	33,032 (3%)
CA	50,806 (5%)
CO	9128 (1%)
CT	12,879 (1%)
DE	7421 (1%)
FL	99,679 (9%)
GA	13,079 (1%)
HI	5284 (<1%)
IA	18,412 (2%)
ID	6538 (1%)
IL	50,471 (5%)
IN	20,789 (2%)
KS	9021 (1%)
KY	21,224 (2%)
LA	7732 (1%)
MA	10,212 (1%)
MD	14,701 (1%)
ME	85 (<1%)
MI	41,774 (4%)
MN	9933 (1%)
MO	14,874 (1%)
MS	11,179 (1%)
MT	6795 (1%)
NC	59,473 (6%)
ND	2704 (<1%)
NE	6775 (1%)
NH	3185 (<1%)
NJ	18,251 (2%)
NM	5922 (1%)
NV	6944 (1%)
NY	73,612 (7%)
OH	56,422 (5%)
OK	22,716 (2%)
OR	18,000 (2%)
PA	54,495 (5%)
RI	3212 (<1%)
SC	25,162 (2%)
SD	4187 (<1%)
TN	33,118 (3%)
TX	59,602 (6%)
UT	119 (<1%)
VA	35,871 (3%)
VT	3534 (<1%)
WA	22,441 (2%)
WI	30,770 (3%)
WV	28,164 (3%)
WY	2206 (<1%)

^1^ SNF: Skilled Nursing Facility; ^2^ ICF: Intermediate Care Facility; ^3^ CMS: Centers for Medicare and Medicaid Services; ^4^ MS-DRG: Medicare Severity Diagnosis Related Group.

**Table 2:** Odds ratios (ORs), 95% confidence intervals (CIs), and *p*-values for univariate and multivariate logistic regression analyses for coding the specificity of a depression principal diagnosis.

		Univariate Analysis			Multivariate Analysis	
Variable	OR	95% CI	*p*	OR	95% CI	*p*
Intercept	-	-	-	0.622	0.315–1.228	0.171
Age (Ref: 85+)						
0–9	4.449	1.964–10.076	<0.001	1.812	0.746–4.403	0.190
10–14	5.179	3.888–6.899	<0.001	2.141	1.483–3.091	<0.001
15–19	4.758	3.605–6.279	<0.001	2.333	1.634–3.331	<0.001
20–24	2.930	2.204–3.895	<0.001	1.982	1.383–2.841	<0.001
25–34	2.292	1.742–3.017	<0.001	1.578	1.113–2.238	0.010
35–44	2.040	1.547–2.690	<0.001	1.459	1.030–2.067	0.034
45–54	2.518	1.904–3.330	<0.001	1.760	1.243–2.490	0.001
55–59	2.346	1.748–3.147	<0.001	1.660	1.160–2.375	0.006
60–64	2.275	1.690–3.063	<0.001	1.699	1.184–2.438	0.004
65–69	2.128	1.567–2.889	<0.001	1.628	1.137–2.331	0.008
70–74	2.207	1.612–3.021	<0.001	1.815	1.258–2.617	0.001
75–79	1.795	1.291–2.497	0.001	1.488	1.015–2.179	0.041
80–84	1.455	1.013–2.091	0.043	1.248	0.821–1.898	0.299
Sex (Ref: Female)						
Male	0.735	0.686–0.786	<0.001	0.760	0.702–0.821	<0.001
Race (Ref: White)						
Asian	1.387	1.095–1.755	0.007	1.221	0.924–1.615	0.161
Black	0.874	0.791–0.965	0.008	0.948	0.841–1.069	0.386
Other	1.008	0.898–1.133	0.889	1.004	0.874–1.153	0.954
Unable to determine	1.222	1.013–1.474	0.037	0.958	0.766–1.197	0.703
Log (Length of Stay)	1.665	1.589–1.745	<0.001	1.820	1.724–1.922	<0.001
Primary Payor (Ref: Medicare traditional)						
Charity/indigent	2.838	1.169–6.891	0.021	1.650	0.599–4.549	0.333
Commercial-indemnity	1.380	1.197–1.591	<0.001	1.394	1.141–1.704	0.001
Direct employer contract	2.167	1.234–3.804	0.007	2.226	1.215–4.077	0.010
Managed care capitated	2.960	1.684–5.203	<0.001	1.332	0.718–2.470	0.363
Managed care non-capitated	1.905	1.686–2.153	<0.001	1.199	1.009–1.425	0.039
Medicaid-managed care capitated	1.035	0.883–1.212	0.675	1.366	1.093–1.707	0.006
Medicaid-managed care non-capitated	1.939	1.706–2.204	<0.001	1.050	0.876–1.260	0.597
Medicaid traditional	1.180	1.030–1.351	0.017	0.838	0.692–1.015	0.071
Medicare-managed care capitated	0.828	0.626–1.095	0.185	1.007	0.717–1.414	0.970
Medicare-managed care non-capitated	1.501	1.275–1.767	<0.001	1.313	1.090–1.582	0.004
Other	2.177	1.700–2.787	<0.001	1.182	0.846–1.650	0.327
Other government payors	2.148	1.700–2.714	<0.001	1.303	0.989–1.718	0.060
Self-Pay	1.460	1.220–1.746	<0.001	1.158	0.922–1.454	0.207
Workers compensation	1.622	0.429–6.135	0.476	3.314	0.753–14.592	0.113
Point of Origin (Ref: Non-healthcare facility)						
Clinic	0.806	0.691–0.940	0.006	1.031	0.826–1.287	0.786
Court/Law enforcement	0.645	0.457–0.911	0.013	0.540	0.368–0.792	0.002
Information not available	1.432	1.213–1.689	<0.001	0.843	0.652–1.090	0.193
Transfer from ambulatory surgery center	1.315	0.477–3.620	0.596	0.820	0.286–2.348	0.711
Transfer from dept unit in same hospital, separate claim	1.757	1.406–2.196	<0.001	1.371	1.056–1.780	0.018
Transfer from health facility	1.199	0.935–1.538	0.153	1.135	0.854–1.509	0.383
Transfer from hospice and under hospice program	0.876	0.079–9.670	0.914	2.441	0.172–34.625	0.510
Transfer from hospital (different facility)	1.487	1.346–1.642	<0.001	1.098	0.968–1.246	0.145
Transfer from SNF ^1^ or ICF ^2^	0.712	0.428–1.186	0.192	0.793	0.444–1.417	0.433
Discharge Status (Ref: Discharged to Home or Self Care)						
Acute inpatient readmission	0.719	0.369–1.402	0.333	1.278	0.612–2.669	0.513
Discharged to home health organization	0.523	0.335–0.816	0.004	1.067	0.646–1.763	0.800
Discharged to hospice-home	0.411	0.149–1.135	0.086	0.516	0.161–1.657	0.266
Discharged to hospice-medical facility	0.432	0.186–1.000	0.050	0.280	0.108–0.721	0.008
Discharged/Transferred to another rehab facility	0.617	0.443–0.859	0.004	0.766	0.522–1.124	0.173
Discharged/Transferred to cancer ctr/children’s hospital	1.919	0.559–6.589	0.301	1.282	0.354–4.646	0.705
Discharged/Transferred to court/law enforcement	0.523	0.335–0.816	0.004	1.067	0.646–1.763	0.800
Discharged/Transferred to critical access hospital	0.360	0.022–5.753	0.470	0.293	0.014–6.096	0.428
Discharged/Transferred to federal hospital	0.360	0.022–5.753	0.470	0.491	0.025–9.699	0.641
Discharged/Transferred to ICF ^2^	0.648	0.414–1.013	0.057	0.459	0.279–0.756	0.002
Discharged/Transferred to long term care hospital	0.899	0.282–2.869	0.858	0.603	0.171–2.126	0.432
Discharged/Transferred to nursing facility	1.239	0.589–2.605	0.572	0.574	0.258–1.277	0.173
Discharged/Transferred to other facility	0.801	0.613–1.048	0.106	1.088	0.806–1.469	0.582
Discharged/Transferred to other health institute not in list	0.658	0.484–0.894	0.007	0.848	0.599–1.201	0.354
Discharged/Transferred to psychiatric hospital	0.719	0.638–0.812	<0.001	0.974	0.843–1.125	0.719
Discharged/Transferred to SNF ^1^	0.352	0.285–0.434	<0.001	0.473	0.362–0.617	<0.001
Discharged/Transferred to swing bed	0.360	0.022–5.753	0.470	0.331	0.017–6.273	0.461
Expired	1.199	0.330–4.360	0.783	1.249	0.295–5.295	0.763
Information not available	0.642	0.334–1.237	0.186	1.867	0.841–4.144	0.125
Left against medical advice	0.371	0.278–0.495	<0.001	0.576	0.414–0.800	0.001
Still a patient-expected to return	0.180	0.016–1.984	0.161	0.160	0.014–1.897	0.146
Count of Procedures	0.945	0.903–0.989	0.015	1.049	0.978–1.125	0.184
CMS ^3^ Fiscal Year (Ref: 2022)						
2023	0.931	0.861–1.007	0.074	0.958	0.877–1.047	0.349
Social Vulnerability Index						
Household characteristics	0.668	0.586–0.760	<0.001	0.640	0.356–1.149	0.135
Housing type and transportation	0.585	0.506–0.676	<0.001	0.731	0.359–1.488	0.388
Overall	0.673	0.589–0.768	<0.001	6.316	0.807–49.465	0.079
Racial and ethnic minority status	0.717	0.623–0.825	<0.001	0.506	0.321–0.798	0.003
Socioeconomic status	0.764	0.671–0.871	<0.001	0.349	0.119–1.020	0.054
COVID-19 Status (Ref: Not identified)						
Positive	0.933	0.819–1.064	0.300	0.943	0.811–1.096	0.441
MS-DRG ^4^ Type (Ref: Medical)						
Surgical	0.135	0.102–0.178	<0.001	0.288	0.204–0.408	<0.001
Teaching Status (Ref: No)						
Not Available	1.038	0.625–1.725	0.885	1.246	0.672–2.311	0.485
Yes	0.596	0.554–0.641	<0.001	0.680	0.572–0.810	<0.001
Academic Status (Ref: No)						
Yes	0.698	0.636–0.765	<0.001	1.465	1.169–1.836	0.001
Rural/Urban Status (Ref: Urban)						
Rural	1.118	1.022–1.223	0.014	1.009	0.854–1.193	0.917
Ownership (Ref: Voluntary non-profit private)						
Government—hospital district/authority	1.530	1.323–1.769	<0.001	0.893	0.716–1.114	0.317
Government—local	1.106	0.920–1.316	0.256	0.512	0.378–0.693	<0.001
Government—state	0.565	0.469–0.681	<0.001	0.408	0.29–0.574	<0.001
Not available	0.776	0.070–8.558	0.836	2.302	0.174–30.375	0.527
Physician	>100	0.000–Inf	0.936	>100	0.000–Inf	0.958
Proprietary	0.547	0.484–0.619	<0.001	0.815	0.650–1.022	0.076
Voluntary non-profit—church	1.135	1.000–1.289	0.050	0.576	0.457–0.727	<0.001
Voluntary non-profit—other	0.688	0.592–0.800	<0.001	0.865	0.688–1.088	0.215
Bed Count (Ref: >400)						
1–50	1.389	0.995–1.941	0.054	0.979	0.615–1.557	0.928
51–100	1.386	1.225–1.568	<0.001	0.914	0.714–1.171	0.477
101–150	0.276	0.223–0.342	<0.001	0.471	0.352–0.628	<0.001
151–200	1.628	1.361–1.947	<0.001	1.728	1.340–2.229	<0.001
201–250	0.992	0.892–1.104	0.889	0.936	0.775–1.132	0.497
251–300	1.674	1.502–1.865	<0.001	1.250	1.035–1.510	0.021
301–350	1.235	1.088–1.402	0.001	1.407	1.110–1.783	0.005
351–400	1.040	0.914–1.182	0.553	1.266	1.002–1.600	0.048
Hospital Case Mix Index	0.881	0.780–0.996	0.043	0.836	0.637–1.097	0.196
State Abbreviation (Ref: NY)						
AK	1.086	0.302–3.909	0.899	1.988	0.450–8.773	0.364
AL	0.724	0.345–1.522	0.394	0.871	0.386–1.965	0.740
AR	1.037	0.751–1.430	0.827	3.641	2.073–6.395	<0.001
AZ	0.269	0.196–0.368	<0.001	0.402	0.264–0.614	<0.001
CA	1.181	0.870–1.605	0.286	1.995	1.393–2.857	<0.001
CO	0.603	0.255–1.429	0.251	1.037	0.409–2.628	0.939
CT	0.996	0.392–2.529	0.993	1.145	0.422–3.104	0.790
DE	1.150	0.607–2.178	0.668	1.600	0.803–3.187	0.181
FL	2.118	1.654–2.711	<0.001	2.652	1.895–3.712	<0.001
GA	1.159	0.590–2.276	0.669	1.760	0.835–3.711	0.138
HI	2.106	1.625–2.730	<0.001	2.883	1.927–4.313	<0.001
IA	1.791	1.014–3.163	0.045	2.056	1.083–3.904	0.028
ID	1.563	0.876–2.788	0.131	1.819	0.888–3.723	0.102
IL	6.770	4.882–9.389	<0.001	5.792	3.905–8.593	<0.001
IN	2.325	1.339–4.037	0.003	2.956	1.583–5.519	0.001
KS	3.645	2.349–5.655	<0.001	4.520	2.686–7.606	<0.001
KY	1.970	1.195–3.246	0.008	2.527	1.368–4.667	0.003
LA	4.918	3.067–7.887	<0.001	3.168	1.744–5.754	<0.001
MA	1.320	0.708–2.464	0.383	1.138	0.565–2.291	0.718
MD	2.367	1.637–3.423	<0.001	2.499	1.567–3.985	<0.001
ME	0.724	0.045–11.66	0.82	0.692	0.041–11.753	0.799
MI	1.696	1.118–2.571	0.013	2.822	1.724–4.619	<0.001
MN	2.858	1.664–4.911	<0.001	3.433	1.916–6.152	<0.001
MO	3.899	2.088–7.280	<0.001	7.573	3.844–14.917	<0.001
MS	0.800	0.417–1.536	0.503	1.041	0.509–2.130	0.911
MT	2.685	2.004–3.596	<0.001	1.520	0.981–2.355	0.061
NC	3.280	2.553–4.215	<0.001	4.336	3.099–6.067	<0.001
ND	3.352	2.351–4.781	<0.001	3.286	1.912–5.648	<0.001
NE	2.723	2.054–3.611	<0.001	3.339	2.092–5.327	<0.001
NH	0.483	0.080–2.921	0.428	0.263	0.040–1.714	0.163
NJ	0.210	0.152–0.289	<0.001	0.247	0.170–0.358	<0.001
NM	0.840	0.475–1.486	0.549	1.046	0.536–2.041	0.895
NV	0.661	0.354–1.234	0.194	1.188	0.584–2.416	0.635
OH	2.763	2.178–3.505	<0.001	3.738	2.682–5.209	<0.001
OK	2.525	1.993–3.198	<0.001	2.917	2.029–4.192	<0.001
OR	1.673	1.036–2.700	0.035	1.543	0.890–2.672	0.122
PA	0.623	0.500–0.776	<0.001	0.673	0.497–0.911	0.010
RI	0.921	0.711–1.194	0.536	0.671	0.429–1.049	0.080
SC	1.798	1.367–2.365	<0.001	1.575	1.085–2.285	0.017
SD	3.331	1.241–8.940	0.017	4.060	1.423–11.586	0.009
TN	1.977	1.210–3.232	0.007	2.223	1.269–3.896	0.005
TX	6.753	5.012–9.099	<0.001	7.051	4.861–10.227	<0.001
UT	0.724	0.045–11.660	0.820	0.569	0.030–10.809	0.707
VA	1.034	0.665–1.609	0.880	1.535	0.928–2.538	0.095
VT	>100	0.000–Inf	0.921	>100	0.000–Inf	0.918
WA	1.228	0.811–1.859	0.332	1.755	1.059–2.909	0.029
WI	3.911	3.026–5.056	<0.001	3.042	2.129–4.345	<0.001
WV	2.286	1.696–3.081	<0.001	1.513	1.017–2.251	0.041
WY	0.677	0.393–1.167	0.160	0.696	0.362–1.341	0.279

^1^ SNF: Skilled Nursing Facility; ^2^ ICF: Intermediate Care Facility; ^3^ CMS: U.S. Centers for Medicare and Medicaid Services; ^4^ MS-DRG: Medicare Severity Diagnosis Related Group.

**Table 3:** Odds ratios (ORs), 95% confidence intervals (CIs), and *p*-values for the univariate and multivariate logistic regression analyses for coding the specificity of depression-related secondary diagnoses.

		Univariate Analysis			Multivariate Analysis	
Variable	OR	95% CI	*p*	OR	95% CI	*p*
Intercept	-	-	-	0.026	0.024–0.029	<0.001
Age (Ref: 85+)						
0–9	1.950	1.195–3.182	0.007	1.729	1.036–2.883	0.036
10–14	6.860	6.345–7.416	<0.001	4.036	3.688–4.416	<0.001
15–19	3.957	3.756–4.169	<0.001	3.002	2.822–3.192	<0.001
20–24	1.806	1.718–1.898	<0.001	1.804	1.704–1.909	<0.001
25–34	1.410	1.356–1.465	<0.001	1.466	1.401–1.535	<0.001
35–44	1.517	1.461–1.575	<0.001	1.554	1.487–1.624	<0.001
45–54	1.447	1.396–1.499	<0.001	1.490	1.430–1.553	<0.001
55–59	1.394	1.342–1.448	<0.001	1.434	1.375–1.496	<0.001
60–64	1.285	1.238–1.333	<0.001	1.316	1.264–1.370	<0.001
65–69	1.283	1.238–1.331	<0.001	1.273	1.225–1.322	<0.001
70–74	1.269	1.224–1.316	<0.001	1.269	1.222–1.317	<0.001
75–79	1.210	1.166–1.257	<0.001	1.211	1.166–1.259	<0.001
80–84	1.117	1.072–1.164	<0.001	1.116	1.070–1.164	<0.001
Sex (Ref: Female)						
Male	1.098	1.082–1.114	<0.001	1.054	1.038–1.071	<0.001
Race (Ref: White)						
Asian	1.148	1.074–1.227	<0.001	0.972	0.905–1.043	0.423
Black	0.921	0.899–0.943	<0.001	0.875	0.853–0.898	<0.001
Other	1.143	1.110–1.177	<0.001	1.032	1.000–1.065	0.051
Unable to determine	1.058	1.009–1.110	0.020	1.039	0.988–1.093	0.139
Log (Length of Stay)	1.177	1.168–1.187	<0.001	1.237	1.225–1.250	<0.001
Primary Payor (Ref: Medicare traditional)						
Charity/indigent	1.853	1.619–2.120	<0.001	1.501	1.305–1.727	<0.001
Commercial-indemnity	1.060	1.024–1.097	0.001	0.846	0.813–0.880	<0.001
Direct employer contract	2.057	1.849–2.288	<0.001	1.497	1.338–1.676	<0.001
Managed care capitated	0.745	0.621–0.894	0.002	0.549	0.455–0.662	<0.001
Managed care non-capitated	1.136	1.109–1.164	<0.001	0.923	0.896–0.951	<0.001
Medicaid-managed care capitated	1.205	1.146–1.268	<0.001	0.951	0.898–1.007	0.086
Medicaid-managed care non-capitated	1.342	1.310–1.375	<0.001	0.979	0.949–1.010	0.187
Medicaid traditional	1.403	1.359–1.448	<0.001	1.011	0.972–1.050	0.593
Medicare-managed care capitated	0.957	0.917–0.999	0.047	1.025	0.979–1.073	0.286
Medicare-managed care non-capitated	1.110	1.088–1.133	<0.001	1.124	1.100–1.148	<0.001
Other	1.061	0.988–1.140	0.106	0.920	0.853–0.993	0.032
Other government payors	0.992	0.938–1.048	0.764	0.846	0.798–0.896	<0.001
Self-Pay	1.489	1.419–1.562	<0.001	1.149	1.089–1.212	<0.001
Worker’s compensation	0.775	0.645–0.933	0.007	0.779	0.646–0.940	0.009
Point of Origin (Ref: Non-healthcare facility)						
Clinic	0.801	0.778–0.825	<0.001	0.914	0.886–0.943	<0.001
Court/Law enforcement	1.482	1.237–1.777	<0.001	0.964	0.786–1.183	0.728
Information not available	0.974	0.913–1.04	0.430	1.375	1.281–1.476	<0.001
Other	1.009	0.786–1.295	0.944	0.906	0.702–1.169	0.448
Transfer from ambulatory surgery center	0.878	0.699–1.102	0.261	1.071	0.849–1.352	0.561
Transfer from dept unit in same hospital, separate claim	1.481	1.357–1.616	<0.001	1.418	1.293–1.555	<0.001
Transfer from health facility	1.083	1.016–1.154	0.014	0.952	0.891–1.018	0.153
Transfer from hospice and under hospice program	1.150	0.556–2.375	0.707	1.213	0.576–2.555	0.612
Transfer from hospital (different facility)	1.003	0.974–1.033	0.828	0.886	0.859–0.915	<0.001
Transfer from SNF ^1^ or ICF ^2^	0.694	0.659–0.732	<0.001	0.754	0.713–0.797	<0.001
Discharge Status (Ref: Discharged to Home or Self Care)						
Acute inpatient readmission	2.125	1.872–2.413	<0.001	1.864	1.634–2.126	<0.001
Discharged to home health organization	0.996	0.975–1.017	0.683	0.996	0.973–1.019	0.732
Discharged to hospice-home	0.941	0.880–1.007	0.077	0.893	0.833–0.957	0.001
Discharged to hospice-medical facility	0.874	0.816–0.937	<0.001	0.880	0.819–0.945	<0.001
Discharged/Transferred to another rehab facility	1.020	0.977–1.064	0.369	0.967	0.924–1.011	0.410
Discharged/Transferred to cancer ctr/children’s hospital	1.919	1.346–2.737	<0.001	1.476	1.021–2.136	0.039
Discharged/Transferred to court/law enforcement	1.466	1.266–1.698	<0.001	1.245	1.059–1.462	0.008
Discharged/Transferred to critical access hospital	1.335	0.822–2.168	0.244	1.331	0.811–2.184	0.257
Discharged/Transferred to federal hospital	0.867	0.545–1.379	0.547	1.020	0.637–1.635	0.933
Discharged/Transferred to ICF ^2^	1.275	1.181–1.377	<0.001	1.127	1.040–1.221	0.004
Discharged/Transferred to long term care hospital	1.013	0.924–1.110	0.781	0.858	0.781–0.944	0.002
Discharged/Transferred to nursing facility	1.596	1.345–1.894	<0.001	1.749	1.469–2.083	<0.001
Discharged/Transferred to other facility	1.136	1.073–1.203	<0.001	1.138	1.074–1.207	<0.001
Discharged/Transferred to other health institute not in list	1.661	1.472–1.874	<0.001	1.632	1.442–1.848	<0.001
Discharged/Transferred to psychiatric hospital	7.779	7.514–8.053	<0.001	6.630	6.387–6.883	<0.001
Discharged/Transferred to SNF ^1^	1.012	0.990–1.034	0.283	0.990	0.965–1.016	0.462
Discharged/Transferred to swing bed	1.048	0.907–1.210	0.525	0.942	0.812–1.092	0.429
Expired	0.876	0.834–0.921	<0.001	0.807	0.766–0.850	<0.001
Information not available	1.439	1.293–1.602	<0.001	2.121	1.885–2.386	<0.001
Left against medical advice	0.970	0.919–1.023	0.263	1.025	0.970–1.083	0.380
Still a patient-expected to return	1.695	0.877–3.275	0.117	1.484	0.754–2.920	0.253
Count of Procedures	1.012	1.010–1.015	<0.001	1.007	1.004–1.009	<0.001
CMS ^3^ Fiscal Year (Ref: 2022)						
2023	1.033	1.016–1.050	<0.001	1.042	1.025–1.060	<0.001
Social Vulnerability Index						
Household characteristics	0.864	0.840–0.889	<0.001	0.776	0.704–0.856	<0.001
Housing type and transportation	0.941	0.914–0.969	<0.001	0.821	0.729–0.925	0.001
Overall	0.732	0.712–0.753	<0.001	2.350	1.672–3.305	<0.001
Racial and ethnic minority status	0.943	0.915–0.971	<0.001	1.111	1.034–1.192	0.004
Socioeconomic status	0.624	0.607–0.642	<0.001	0.529	0.442–0.634	<0.001
COVID-19 Status (Ref: Not identified)						
Positive	0.986	0.959–1.014	0.333	0.929	0.902–0.957	<0.001
MS-DRG ^4^ Type (Ref: Medical)						
Surgical	0.843	0.829–0.858	<0.001	0.855	0.839–0.871	<0.001
Unknown	1.296	0.515–3.266	0.582	1.735	0.679–4.433	0.250
Teaching Status (Ref: No)						
Not Available	1.848	1.762–1.940	<0.001	1.713	1.622–1.810	<0.001
Yes	1.064	1.047–1.082	<0.001	1.177	1.143–1.212	<0.001
Academic Status (Ref: No)						
Yes	1.042	1.022–1.062	<0.001	0.790	0.763–0.819	<0.001
Rural/Urban Status (Ref: Urban)						
Rural	1.081	1.058–1.105	<0.001	1.409	1.371–1.447	<0.001
Ownership (Ref: Voluntary non-profit private)						
Government—federal	0.491	0.378–0.639	<0.001	0.232	0.177–0.304	<0.001
Government—hospital district/authority	1.345	1.309–1.382	<0.001	1.457	1.411–1.505	<0.001
Government—local	0.992	0.953–1.033	0.707	0.987	0.942–1.034	0.577
Government—state	0.782	0.720–0.849	<0.001	1.076	0.981–1.180	0.122
Not available	0.782	0.696–0.88	<0.001	0.835	0.739–0.943	0.004
Physician	0.562	0.425–0.742	<0.001	0.423	0.318–0.563	<0.001
Proprietary	0.698	0.669–0.728	<0.001	0.793	0.757–0.831	<0.001
Voluntary non-profit—church	0.751	0.734–0.768	<0.001	0.846	0.825–0.868	<0.001
Voluntary non-profit—other	0.918	0.890–0.948	<0.001	0.894	0.863–0.926	<0.001
Bed Count (Ref: >400)						
1–50	0.973	0.931–1.017	0.230	0.578	0.547–0.610	<0.001
51–100	0.925	0.894–0.957	<0.001	0.884	0.850–0.920	<0.001
101–150	0.605	0.585–0.626	<0.001	0.571	0.550–0.593	<0.001
151–200	0.967	0.939–0.995	0.021	0.753	0.728–0.779	<0.001
201–250	0.873	0.85–0.896	<0.001	0.885	0.858–0.912	<0.001
251–300	0.735	0.715–0.755	<0.001	0.692	0.670–0.714	<0.001
301–350	0.843	0.822–0.865	<0.001	0.937	0.909–0.965	<0.001
351–400	0.867	0.841–0.893	<0.001	0.875	0.847–0.905	<0.001
Hospital Case Mix Index	0.966	0.942–0.990	0.006	0.873	0.843–0.904	<0.001
State Abbreviation (Ref: NY)						
AK	2.142	1.520–3.017	<0.001	3.013	2.124–4.274	<0.001
AL	0.742	0.647–0.851	<0.001	0.829	0.719–0.954	0.009
AR	0.997	0.899–1.105	0.954	1.091	0.975–1.220	0.130
AZ	1.119	1.049–1.194	0.001	1.121	1.044–1.204	0.002
CA	2.437	2.322–2.557	<0.001	2.778	2.634–2.931	<0.001
CO	1.492	1.355–1.642	<0.001	1.691	1.529–1.869	<0.001
CT	2.142	1.990–2.306	<0.001	2.227	2.059–2.409	<0.001
DE	1.806	1.637–1.991	<0.001	1.503	1.356–1.666	<0.001
FL	1.802	1.723–1.885	<0.001	2.000	1.900–2.104	<0.001
GA	1.592	1.468–1.727	<0.001	1.739	1.594–1.898	<0.001
HI	3.092	2.800–3.416	<0.001	2.786	2.493–3.112	<0.001
IA	6.113	5.803–6.440	<0.001	7.055	6.647–7.488	<0.001
ID	7.100	6.625–7.610	<0.001	10.95	10.09–11.88	<0.001
IL	1.766	1.677–1.859	<0.001	2.278	2.153–2.409	<0.001
IN	1.133	1.050–1.223	0.001	1.420	1.310–1.540	<0.001
KS	1.042	0.932–1.166	0.471	1.167	1.038–1.312	0.010
KY	1.477	1.378–1.583	<0.001	1.717	1.594–1.850	<0.001
LA	2.890	2.661–3.139	<0.001	2.943	2.696–3.212	<0.001
MA	1.699	1.557–1.854	<0.001	1.801	1.642–1.975	<0.001
MD	1.351	1.244–1.467	<0.001	1.540	1.413–1.679	<0.001
ME	1.579	0.639–3.903	0.323	2.124	0.856–5.268	0.104
MI	1.977	1.876–2.083	<0.001	2.502	2.360–2.653	<0.001
MN	9.702	9.158–10.277	<0.001	11.25	10.57–11.98	<0.001
MO	3.475	3.268–3.696	<0.001	3.435	3.212–3.673	<0.001
MS	0.924	0.831–1.028	0.146	0.962	0.862–1.075	0.494
MT	2.855	2.611–3.121	<0.001	3.751	3.400–4.139	<0.001
NC	2.421	2.310–2.538	<0.001	2.409	2.285–2.540	<0.001
ND	1.862	1.586–2.187	<0.001	2.505	2.118–2.962	<0.001
NE	3.548	3.262–3.858	<0.001	5.412	4.944–5.923	<0.001
NH	0.924	0.764–1.117	0.415	1.292	1.064–1.567	0.010
NJ	1.544	1.436–1.661	<0.001	1.749	1.622–1.887	<0.001
NM	4.086	3.763–4.436	<0.001	4.193	3.839–4.580	<0.001
NV	1.645	1.482–1.826	<0.001	2.445	2.184–2.737	<0.001
OH	1.444	1.371–1.522	<0.001	1.733	1.636–1.835	<0.001
OK	2.270	2.135–2.412	<0.001	2.311	2.159–2.474	<0.001
OR	3.359	3.168–3.562	<0.001	4.143	3.884–4.419	<0.001
PA	3.021	2.885–3.165	<0.001	3.723	3.536–3.919	<0.001
RI	1.033	0.849–1.258	0.743	1.381	1.125–1.695	0.002
SC	1.896	1.783–2.015	<0.001	2.065	1.933–2.206	<0.001
SD	4.671	4.264–5.116	<0.001	5.327	4.829–5.876	<0.001
TN	1.037	0.970–1.108	0.286	1.28	1.191–1.376	<0.001
TX	1.898	1.807–1.993	<0.001	2.120	2.006–2.241	<0.001
UT	1.808	0.881–3.710	0.107	1.775	0.841–3.748	0.132
VA	1.344	1.266–1.427	<0.001	1.383	1.295–1.477	<0.001
VT	0.644	0.521–0.797	<0.001	0.639	0.512–0.797	<0.001
WA	2.034	1.912–2.164	<0.001	2.837	2.653–3.035	<0.001
WI	3.424	3.253–3.603	<0.001	4.216	3.985–4.461	<0.001
WV	1.624	1.527–1.727	<0.001	2.011	1.878–2.155	<0.001
WY	2.462	2.115–2.865	<0.001	3.007	2.565–3.524	<0.001

^1^ SNF: Skilled Nursing Facility; ^2^ ICF: Intermediate Care Facility; ^3^ CMS: U.S. Centers for Medicare and Medicaid Services; ^4^ MS-DRG: Medicare Severity Diagnosis Related Group.

## Data Availability

The datasets presented in this article are not readily available because they are the property of Premier, Inc. Requests to access the datasets should be directed to Premier, Inc. via pinc-ai.com.

## References

[1] Hirsch J.A., Nicola G.N., McGinty G., Liu R.W., Barr R.M., Chittle M.D., Manchikanti L. (2016). ICD-10: History and Context. Am. J. Neuroradiol..

[2] Centers for Disease Control and Prevention (CDC) (2015). International Classification of Diseases, (ICD-10-CM/PCS) Transition-Background. CDC. https://www.cdc.gov/nchs/icd/icd10cm_pcs_background.htm.

[3] Boyd A.D., Li J., Burton M., Jonen M., Gardeux V., Achour I., Luo R.Q., Zenku I., Bahroos N., Brown S.B. (2013). The Discriminatory Cost of ICD-10-CM Transition between Clinical Specialties: Metrics, Case Study, and Mitigating Tools. J. Am. Med. Inform. Assoc..

[4] Grasso M.A., Dezman Z.D.W., Jerrard D.A. (2022). Coding Disparity and Specificity during Emergency Department Visits after Transitioning to the Tenth Version of the International Classification of Diseases. AMIA Annual Symposium Proceedings.

[5] Zegan J. Improving Specificity in ICD-10 Diagnosis Coding. American Health Information Management Association (AHIMA). https://library.ahima.org/doc?oid=302473.

[6] Rangachari P. (2007). Coding for Quality Measurement: The Relationship between Hospital Structural Characteristics and Coding Accuracy from the Perspective of Quality Measurement. Perspect. Health Inf. Manag..

[7] Department of Health and Human Services Information and Resources for Submitting Correct ICD-10 Codes to Medicare. Medicare Learning Network (MLN) Matters 2014: SE1518. https://www.hhs.gov/guidance/sites/default/files/hhs-guidance-documents/SE1518.pdf.

[8] American Hospital Association (AHA) (2013). Using the X-ray Report for Specificity. AHA Coding Clinic for ICD-10-CM and ICD-10-PCS (First Quarter 2013).

[9] American Hospital Association (AHA) (2014). Use of Imaging Reports for Greater Specificity. AHA Coding Clinic for ICD-10-CM and ICD-10-PCS (Third Quarter 2014).

[10] American Hospital Association (AHA) (2016). Use of X-ray to Determine Site of Pain. AHA Coding Clinic for ICD-10-CM and ICD-10-PCS (Fourth Quarter 2016).

[11] Mendez C.M., Harrington D.W., Christenson P.D., Spellberg B. (2014). Impact of Hospital Variables on Case Mix Index as a Marker of Disease Severity. Popul. Health Manag..

[12] O’Malley K.J., Cook K.F., Price M.D., Wildes K.R., Hurdle J.F., Ashton C.M. (2005). Measuring Diagnoses: ICD Code Accuracy. Health Serv. Res..

[13] Horsky J., Drucker E.A., Ramelson H.Z. (2017). Accuracy and Completeness of Clinical Coding Using ICD-10 for Ambulatory Visits. AMIA Annual Symposium Proceedings.

[14] Beam K.S., Lee M., Hirst K., Beam A., Parad R.B. (2021). Specificity of International Classification of Diseases Codes for Bronchopulmonary Dysplasia: An Investigation Using Electronic Health Record Data and a Large Insurance Database. J. Perinatol..

[15] Quan H., Li B., Saunders D.L., Parsons G.A., Nilsson C., Alibhai A., Ghali W.A. (2008). Assessing Validity of ICD-9-CM and ICD-10 Administrative Data in Recording Clinical Conditions in a Unique Dually Coded Database. Health Serv. Res..

[16] Rios N.G., Oldiges P.E., Lizano M.S., Daucet-Wadford D.S., Quick D.L., Martin J.K., Korvink M., Gunn L.H. (2022). Modeling Coding Intensity of Procedures in a U.S. Population-Based Hip/Knee Arthroplasty Inpatient Cohort Adjusting for Patient- and Facility-Level Characteristics. Healthcare.

[17] Mishra R., Verma H., Aynala V.B., Arredondo P.R., Martin J.K., Korvink M., Gunn L.H. (2022). Diagnostic Coding Intensity among a Pneumonia Inpatient Cohort Using a Risk-Adjustment Model and Claims Data: A U.S. Population-Based Study. Diagnostics.

[18] Lee B., Wang Y., Carlson S.A., Greenlund K.J., Lu H., Liu Y., Croft J.B., Eke P.I., Town M., Thomas C.C. (2023). National, State-Level, and County-Level Prevalence Estimates of Adults Aged ≥ 18 Years Self-Reporting A Lifetime Diagnosis of Depression—United States, 2020. MMWR Morb. Mortal. Wkly. Rep..

[19] Cuncic A., Block D.B. What Are the ICD-10 Criteria for Depression?. https://www.verywellmind.com/icd-10-criteria-for-depression-5308497.

[20] 2024 ICD-10-CM Diagnosis Code F32.9: Major Depressive Disorder, Single Episode, Unspecified. https://www.icd10data.com/ICD10CM/Codes/F01-F99/F30-F39/F32-/F32.9.

[21] Torres F. What Is Depression? American Psychiatric Association. October 2020. https://www.psychiatry.org/patients-families/depression/what-is-depression.

[22] PINC AI Applied Sciences (2023). PINC AI Healthcare Database White Paper: Data That Informs and Performs.

[23] Centers for Disease Control and Prevention (CDC), Agency for Toxic Substances and Disease Registry (2020). CDC SVI Documentation. https://www.atsdr.cdc.gov/placeandhealthsvi/documentation/SVI_documentation_2020.html.

[24] Centers for Medicare & Medicaid Services, Office of the Actuary, National Health Statistics Group (2022). National Health Expenditure Data: Health Expenditures by State of Residence. https://www.cms.gov/data-research/statistics-trends-and-reports/national-health-expenditure-data/state-residence.

[25] Higashi T., Shekelle P.G., Solomon D., Knight E.L., Roth C.P., Chang J.T., Kamberg C., MacLean C., Young R., Adams J.L. (2004). Quality of Health Care Received by Older Adults.

[26] Richardson A., Allen J.A., Xiao H., Vallone D. (2012). Effects of Race/Ethnicity and Socioeconomic Status on Health Information-Seeking, Confidence, and Trust. J. Health Care Poor Underserved..

[27] Vyas C.M., Donneyong M., Mischoulon D., Chang G., Gibson H., Cook N.R., Manson J.E., Reynolds III C.F., Okereke O.I. (2020). Association of Race and Ethnicity with Late-Life Depression Severity, Symptom Burden, and Care. JAMA Netw. Open.

[28] United States Census Bureau Age-Sex Pyramid for the United States. https://www.census.gov/library/visualizations/interactive/age-sex-pyramid-for-the-united-states.html.

[29] Johnson E.K., Wojtesta M.A., Crosby S.W., Duber H.C., Jun E., Lescinsky H., Nguyen P., Sahu M., Thomson A., Tsakalos G. (2022). Varied Health Spending Growth Across US States Was Associated with Incomes, Price Levels, and Medicaid Expansion, 2000–2019. Health Aff..

[30] Vyas D.A., Eisenstein L.G., Jones D.S. (2020). Hidden in Plain Sight—Reconsidering the Use of Race Correction in Clinical Algorithms. N. Engl. J. Med..

